# Robot-Assisted Multiquadrant Operation: Proctocolectomy and Abdominoperineal Resection Using the Da Vinci Xi for Familial Adenomatous Polyposis With Rectal Carcinoma

**DOI:** 10.7759/cureus.85107

**Published:** 2025-05-30

**Authors:** Turgay Kalinov, Aleksandar Zlatarov, Nikola Kolev, Krasimir D Ivanov

**Affiliations:** 1 General and Operative Surgery, Faculty of Medicine, Medical University of Varna, Varna, BGR; 2 General and Operative Surgery, Medical University "Prof. Dr. Paraskev Stoyanov", Varna, BGR; 3 General and Operative Surgery, University Hospital St. Marina, Varna, BGR

**Keywords:** abdominoperineal resection, cancer, familial adenomatous polyposis, fap, proctocolectomy, rectal cancer, rectum, robot-assisted resection

## Abstract

Familial adenomatous polyposis (FAP) is a rare inherited disorder that is characterized by the formation of multiple adenomatous polyps in the colon that are precancerous. Diagnosis of the disease is of utmost importance in patients with a history of FAP, and prophylactic fibrocolonoscopies should be performed as early as the teenage years. This rare but significant genetic condition usually manifests in late adolescence or early adult years. The clinical case highlights the importance of a multidisciplinary approach and the need for early diagnosis and treatment in patients with FAP. The da Vinci Xi robotic system (Intuitive Surgical, CA, USA) has been successfully applied in multiquadrant operations such as total colectomy with abdominoperineal extirpation of the rectum.

We present the case of a 38-year-old man admitted to our clinic, the First Clinic of Surgery in University Hospital Saint Marina, Varna, Bulgaria, in late 2022 with complaints of blood in stool and abnormal defecation rhythm. The fibrocolonoscopy found a tumor formation at 4 cm of the anorectal junction, a second tumor formation at 10 cm, and multiple colonic polyposis. Biopsies taken from the suspected areas confirmed the presence of two rectal carcinomas and adenomatous polyposis in the remaining areas. Neoadjuvant radiotherapy and chemotherapy with capecitabine 1600 mg were performed. Persistent carcinoma was an indication for performing robot-assisted proctocolectomy combined with abdominoperineal resection of the rectum. The robotic platform we used was the da Vinci Xi, a single-docking patient platform with three boom repositioning. The patient was followed for 24 months postoperatively. Recovery was uneventful, with no major complications. The terminal ileostomy functioned well, and the patient adapted successfully to stoma care. During follow-up, no local or systemic recurrence of the oncological disease was observed, and surveillance imaging remained clear throughout the period.

This clinical case provides an important example of the multidisciplinary approach in the diagnosis and treatment of FAP. Neoadjuvant therapy, including radiotherapy and chemotherapy followed by robot-assisted surgery, demonstrates the significant potential of modern treatment approaches. Robot-assisted proctocolectomy is a relatively new approach whose advantages and disadvantages are yet to be explored. Collaboration between gastroenterologists, oncologists, radiologists, and surgeons is essential for the comprehensive management of such complex cases. Finally, this clinical case highlights the need for long-term surveillance of patients with FAP for early detection and management of potential recurrences or new disease.

## Introduction

Familial adenomatous polyposis (FAP) is a rare hereditary condition marked by the development of numerous adenomatous polyps in the colon, representing a precancerous state. Early diagnosis is crucial, particularly in individuals with a family history of FAP, and preventive fibrocolonoscopies are recommended beginning in adolescence. Though uncommon, this important genetic disorder typically presents in late teenage years or early adulthood. FAP is caused by mutations in the APC gene, which plays a key role in regulating cell growth and apoptosis. The presence of multiple polyps significantly increases the risk of developing colorectal cancer if the polyps are not treated promptly [1].

FAP occurs in individuals of all ethnic groups and is usually diagnosed between the ages of 10 and 30, although the first symptoms may appear earlier. Surveillance and early diagnosis are essential, as prevention and control of the disease can significantly reduce the risk of colorectal cancer.

Prevention and treatment of FAP require regular surveillance and screening, especially in individuals with a family history of the disease. Screening usually begins in adolescence with a colonoscopy to identify and remove polyps before they become malignant. Genetic counseling is also important to assess risk and inform families of the potential risk of inheriting the disease.

Treatment of FAP usually requires surgical intervention, which involves performing a proctocolectomy with or without a J-pouch ileo-anal anastomosis. Prophylactic colectomy is the standard approach to prevent colon cancer in these patients. However, the decision on the timing and extent of surgery depends on the number, size, and distribution of polyps, as well as the patient's general health. In addition, current medical research is exploring opportunities for medical treatment and observation that may offer alternative or complementary approaches in the management of this condition [2]. 

If the diagnosis is not made on time, the first manifestation of FAP is associated with the presence of rectorrhagia, which may be triggered by the development of colon and/or rectal cancer. This changes the treatment algorithm, involving the conduct of neoadjuvant and/or adjuvant radiotherapy and chemotherapy. In the presence of low-grade rectal carcinoma, surgery can be extended to perform a proctocolectomy combined with abdomino-perineal extirpation of the rectum.

Thanks to the development of minimally invasive surgery in recent years, the surgical intervention can be performed not only by a conventional approach but also by laparoscopic or robot-assisted surgery. Proctectomy, especially if combined with extirpation of the rectum, requires dissection, mobilization, and ligation of vessels in several abdominal quadrants.

Because of the structure of older robotic da Vinci systems (Intuitive Surgical, CA, USA) and the need to reposition the patient console or use a hybrid laparoscopic-robotic technique, they are not the preferred method of choice for multiquadrant operations. The functionality of the patient console in da Vinci Xi systems is unique and only found in this robot model due to the specific technical features it has. Thanks to the possibility of 360-degree rotation of the support arms (boom), as well as the possibility of adjustment in the angle of operation of the surgical arms (patient clearance), access to the four abdominal quadrants can be achieved with a single positioning of the patient console around the patient [3].

## Case presentation

We present the case of a 38-year-old man admitted to the First Clinic of Surgery in University Hospital Saint Marina, Varna, Bulgaria, in late 2022 with complaints of blood in stool and abnormal defecation rhythm. The patient reported no comorbidities or previous surgical interventions. He does not smoke, abuse alcohol, or use drugs.

The fibrocolonoscopy found a tumor formation at 4 cm of the anorectal junction, a second tumor formation at 10 cm, and multiple colonic polyposis. Biopsies taken from the suspected areas confirmed the presence of two rectal carcinomas and adenomatous polyposis in the remaining areas. After further computed tomography (CT) of the abdomen, magnetic resonance imaging (MRI) of the pelvis, and endorectal ultrasonography, the stage was determined as T3aN0. PET/CT showed no evidence of distant organ or lymph node metastases.

After discussion at the oncology committee, a decision was made to conduct neoadjuvant radiotherapy and chemotherapy. Neoadjuvant radiotherapy and chemotherapy with capecitabine 1600 mg were performed.

**Figure 1:**
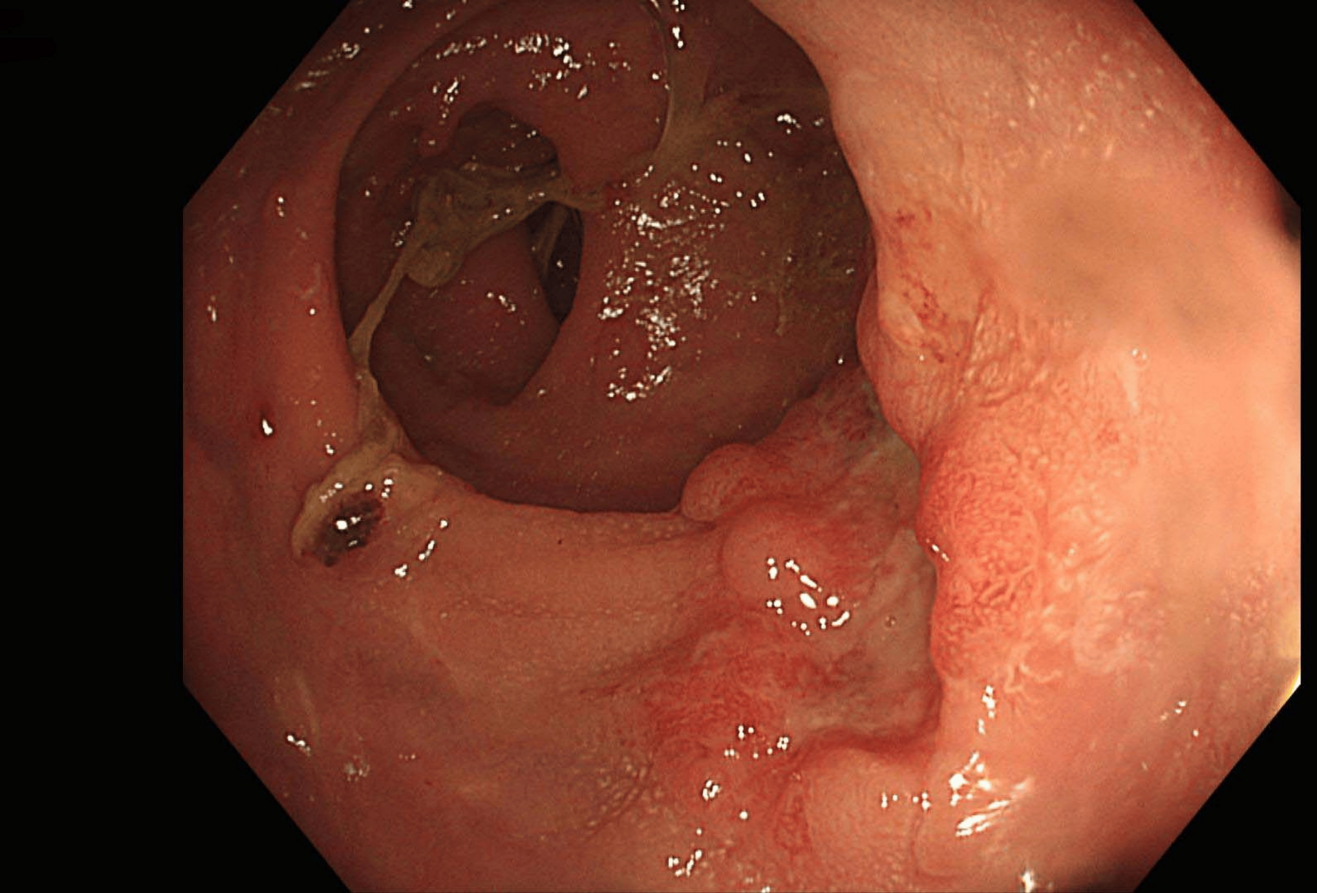
Tumor process at 4 cm with a partial response to the therapy

**Figure 2:**
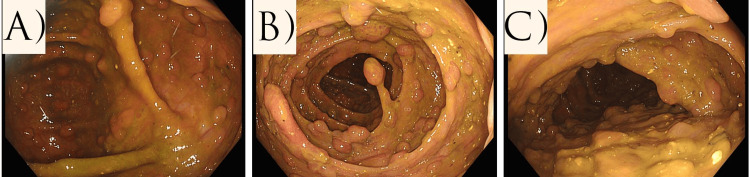
Colonic polyposis We see multiple colon polyposis along the entire length of the colon. A: colon descendants; B: colon transversum; C: colon ascendens

One month after completion of the neoadjuvant therapy, the patient underwent restaging endoscopic and imaging examinations. Fiberoptic colonoscopy showed a partial reduction in the size of the tumor process at 4 cm, with persistent carcinoma cells, while the tumor process at 10 cm showed a complete response to treatment (Figure 1). The MRI performed assessed the response from therapy for the 4 cm tumor as tumor regression grading (TRG) 1-2 (almost complete response by modified Ryan scheme). Colonic polyposis was without significant dynamics compared to the initial finding (Figure 2).

**Figure 3:**
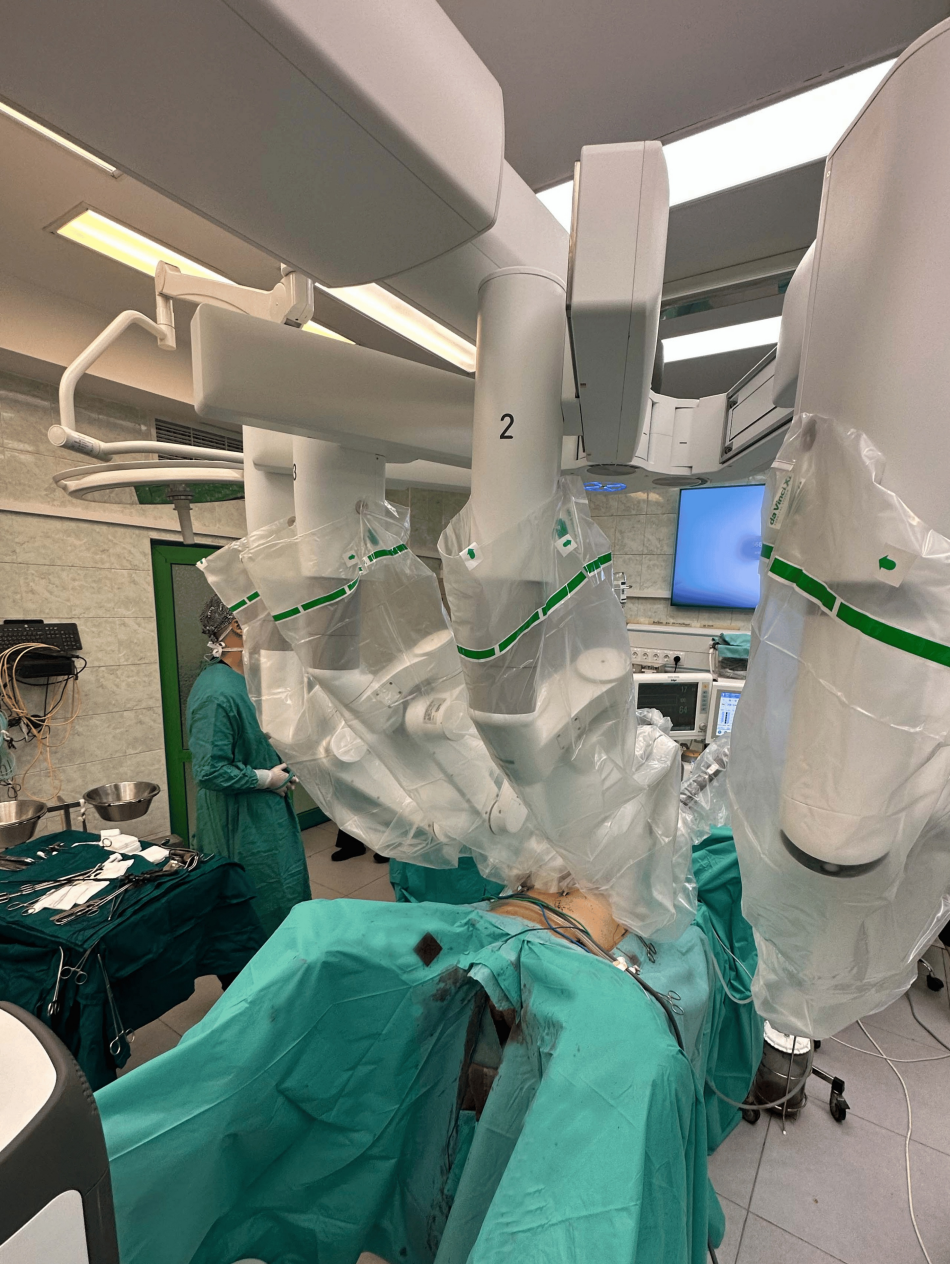
Positioning of the da Vinci Xi system Positioning the patient console between the patient's legs.

Persistent carcinoma was an indication for performing robot-assisted proctocolectomy combined with abdominoperineal resection of the rectum. The robotic platform we used was the da Vinci Xi, a single-docking patient platform with three boom repositioning. For instrument insertion, we used six ports sequentially for the individual abdominal quadrants. The patient was placed in the supine position on an antecubital mattress with legs abducted and extended (Figure 3).

**Figure 4:**
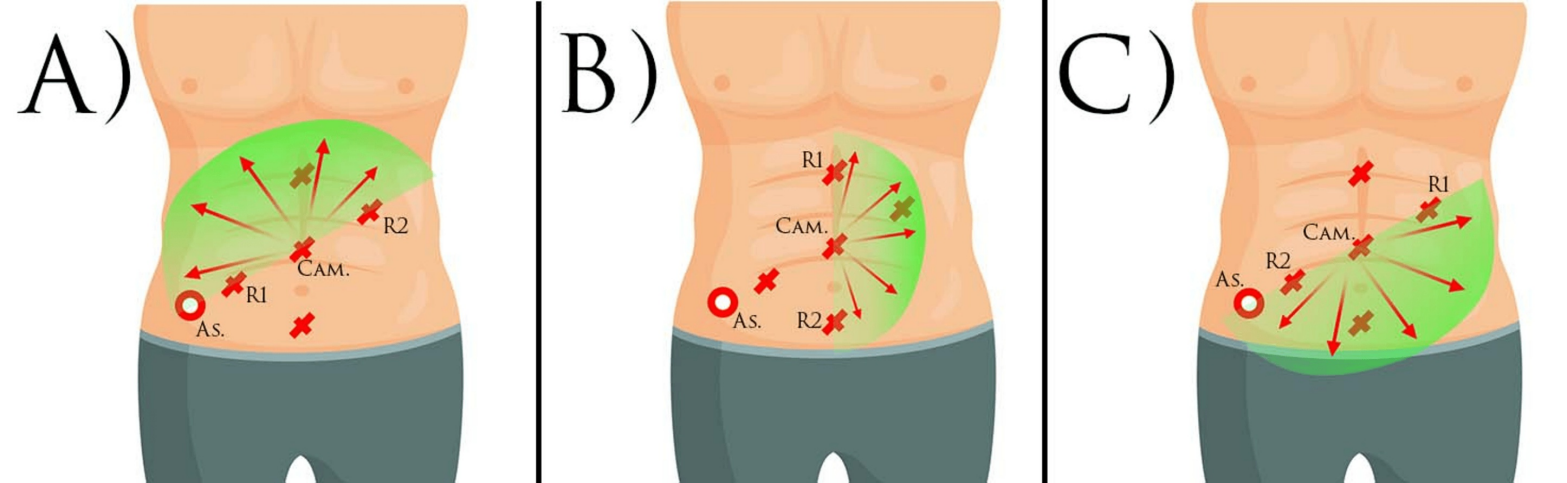
Port positioning Original figure created by the author. This figure presents the positioning of the boom of the robot. A: operation on the colon ascendens and transverse colon; B: operation on the descending colon; C: operation in the small pelvis and rectum. As: assistant port; R1: robot arm 1; R2: robot arm 2; Cam: camera port

Surgery began with the release and disruption of the terminal ileum, along with the ligation of the ileocolic artery and the ileocolic vein. This step allows for technically better visual access in the subsequent steps, which include ligation and cutting of the artery and vein colica dextra. Colon ascendens, hepatic flexure, and colon transversum were mobilized (Figure 4A). The feeding vessels were also selectively ligated and cuted. After rotation of the boom for a better view of the left part of the abdominal cavity, the hepatic flexure and colon descendens were mobilized, and the feeding vessel was divided and cuted (Figure 4B). Due to the absence of evidence of carcinoma along the colonic course, D1 lymph node dissection was performed. The second repositioning of the boom, with guidance in the caudal direction, allowed mobilization of the sigmoid colon and rectum (Figure 4C). The inferior mesenteric artery and vein were tied off above, allowing the rectum to be transected to the levator ani muscles, and a total mesorectal excision along with D2 lymph node dissection was done. The operation was completed with conventional perineal extirpation according to Miles. Due to the lack of sufficient free peritoneum, the pelvic floor was closed by fixing a polypropylene mesh. The passage led out in the form of a definitive ileostomy through the orifice used for the AirSeal port (AirSeal® System, CONMED Corporation, Florida, USA) during surgery.

**Figure 5:**
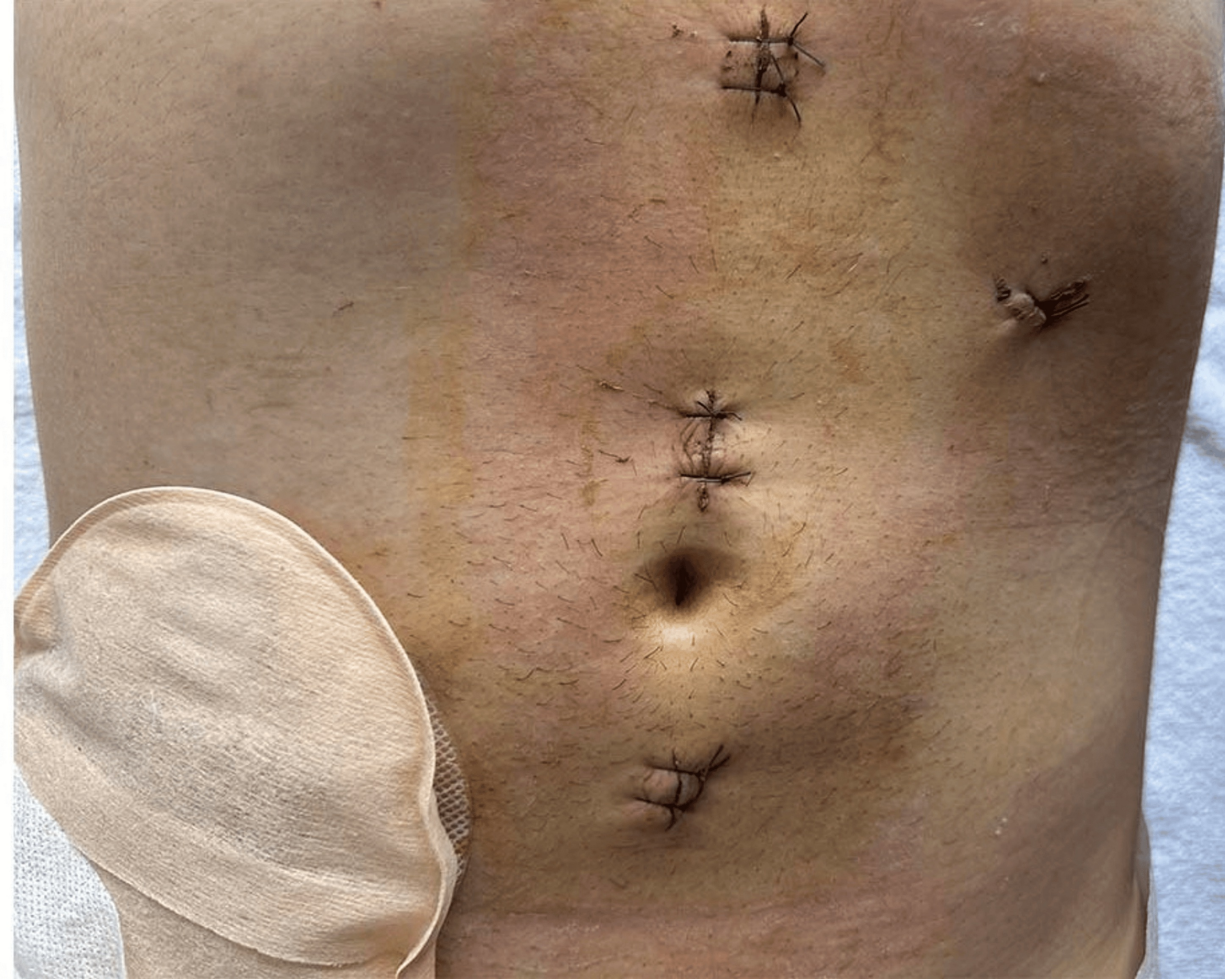
Cosmetic effect

Intraoperative antibiotic prophylaxis was carried out with 2x2 grams of cefazolin powder dissolved in 20 ml of physiological solution intravenously and metronidazole 2x500 mg/100 ml intravenously. Postoperative antibiotic treatment was continued for five days in doses of 3x2 g cefazolin and 3x500mg metronidazole. In the first 24 hours after surgery, 4x1 analgin and 2x½ lidol were used for analgesia, then 3x1 analgin was used until the fourth postoperative day. Anticoagulant therapy with a single daily dose of fraxiparine 0.6 was applied for seven days postoperatively. The operative time was 350 min, and the blood loss was about 100 ml. In the early postoperative period, the patient was found to have subcutaneous emphysema spreading from the clavicle to the inguinal folds, which resolved without complications in five days. The perineal wound was swabbed for 48 hours, after which the compresses were removed. The cosmetic effect can be seen in Figure 5.

Within the first 24 hours, the patient was ambulated and started drinking liquids with an active ileostomy. At the 48th hour, the patient started the intake of liquid and solid food. There were no complications on the surgical wound side, and the patient was discharged on the seventh postoperative day.

**Figure 6:**
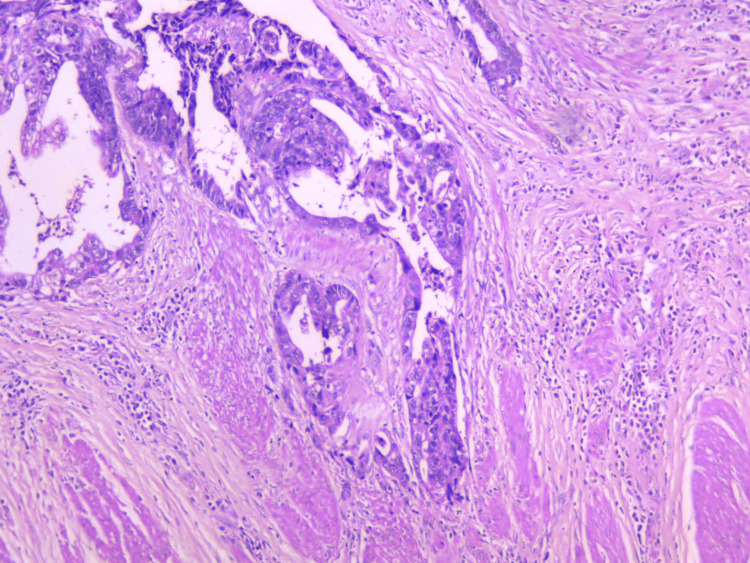
Invasion of muscularis propria from infiltrative tumor acini

**Figure 7:**
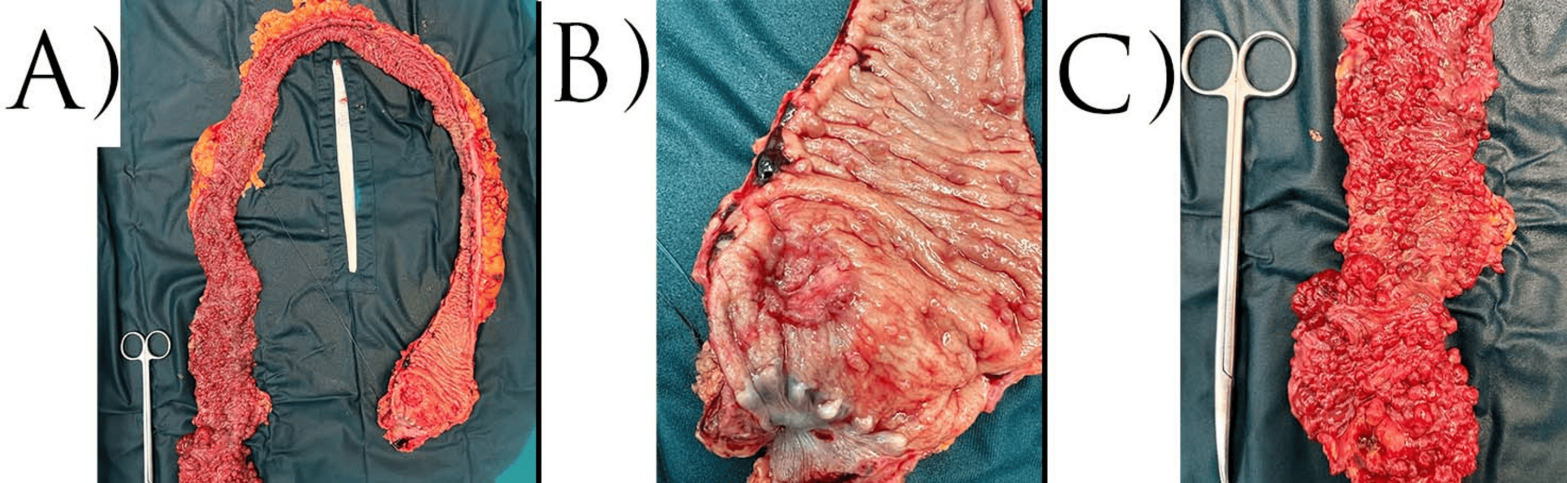
Macroscopic view of the colon A: multiple polyposis throughout the colon; B: carcinoma of the rectum; C: large ascending colon polyp with suspicion of malignancy

The histological examination of the resected colonic specimen confirmed the presence of multiple adenomatous polyps throughout the colon, as well as an adenocarcinoma of the rectum, staged as ypT2N1c, G2, R0, LVI (+). The histological result can be seen in Figure 6. Photomicrograph showing invasion of muscularis propria from infiltrative tumor acini, H&E, x10 (Figure 6). The tumor response to radiotherapy was graded as two out of three according to the modified Ryan scheme. Macroscopic examination demonstrated the presence of low rectal carcinoma as well as multiple colonic polyposis, most of them found in the colon ascendens and colon transversum (Figure 7).

## Discussion

FAP is a rare, inherited condition characterized by the development of multiple precancerous polyps in the colon. This disease poses a significant risk for the development of colorectal cancer, with the risk reaching almost 100% in the absence of treatment. The average age of cancer diagnosis in patients with FAP is 39 years. Polyps usually begin to develop in the teens or early 20s, and they become larger and more problematic as people age [4].

FAP is caused by mutations in the APC gene that disrupt the function of the protein produced by the gene, allowing cells to grow in an uncontrolled manner. About 25% of cases of FAP are due to new mutations that occur during the embryonic period, in which the patient has no family history of FAP [5].

In addition to the colon, FAP can lead to the development of polyps and other anomalies in various organs, including the stomach, duodenum, thyroid, bones, and brain. This disease can manifest in various clinical forms, such as classic FAP, Gardner's syndrome, attenuated FAP, and Turcot syndrome, each of which has its own specific characteristics and associated risks.

The clinical case we are presenting is interesting because the patient reported no comorbidities or previous surgical interventions and no known risk factors such as tobacco, alcohol, or drug use. This highlights the fact that FAP can occur even in individuals without a clear family history of the disease, especially in the presence of novel mutations.

Robot-assisted surgery is a method that offers increased precision and potentially faster recovery. One of the advantages of the da Vinci Xi system is the ability to reposition the surgical arms in a variety of ways, which optimizes the surgical workspace and facilitates access to different quadrants without the need to reposition the patient and patient console. This is made possible by the unique structure of the patient console, which is composed of a horizontal boom with 360° rotation capability and multi-position joints on each of the four robotic arms. The identical structure of each arm allows exchanging the instruments and the endoscope in the most comfortable and desirable way possible. These capabilities are only available for the da Vinci Xi system, which makes it a significantly more convenient choice of operation for multiquadrant surgeries, unlike older systems.

Each surgical intervention has specific challenges and requires additional preparation time before the surgery itself, such as the need for accurate positioning of the patient's cart and surgical instruments. For each surgical intervention, the positioning of the endoscopic camera, robotic arms, and assistant port is specific to the abdominal quadrant in which the surgery will be performed. The challenge with total proctocolectomy is the need to work in four different abdominal quadrants. This requires a specific combination of port localization. In our case, we chose two lines, which, by placing the ports, give us the possibility, after each repositioning of the boom, to work in the different abdominal quadrants, with a centrally located suprapubic port for the camera. By docking along the diagonal line in the cranial direction between the spina iliaca superior and the left subdiaphragmatic, in the cranial direction, we have the possibility of working on the right abdominal flank, right subhepatic space, and epigastrium, while docking in the caudal direction along the same line, we can work on the lower third of the left abdomen, sigmoid space, and small pelvis. By docking along the xiphopubic line with a right or left orientation, we have full access to the right or left abdominal flank, respectively, with right/left colon mobilization with flexor mobilization (Figure 4). Our experience shows that the described port localization reduces the number of rotations and repositions of the boom by one.

One of the benefits of Xi systems, over older robotic systems, is the unlimited ability to use the ports. Whereas with previous systems, the endoscopic camera could be placed on one specific port and robotic arm, with the Xi system, the camera can be placed on any arm and port. Typically, the optimal distance between ports that allows freedom of movement is 8 cm. Due to the additional robotic joint on each arm and the possibility of patient clearance adjustment, a larger working angle is allowed, and the risk of overlapping is minimal. For the assistant port, we used a 12 mm AirSeal port (Figure 4 (As)) placed along the lines of the other ports. Through it, the first assistant had access to all quadrants during the operation.

Due to the patient's small abdominal cavity and limited working space, we used three robotic arms and a 30-degree endoscopic camera. The robotic instruments we used were monopolar scissors, fenestrated bipolar forceps, and Ultracision Harmonic Ace (Ethicon, Inc., NJ, USA). For passage interruption, we used a linear echelon with 80 mm green filler.

It is difficult to determine a specific percentage of operative time associated with this preparation, but it is important to note that a high degree of precision in positioning can significantly improve minimally invasive treatment options and the final outcome for the patient. In our case, the preparation time for the robotic system and docking to the patient typically takes about 20 minutes.

The benefits of robot-assisted surgical interventions could highlight the classical advantages of the minimally invasive approach: less trauma, less blood loss, need for less anesthesia, rapid verticalization, refeeding, and dehospitalization. The advantages over the laparoscopic approach are the finer and more detailed tissue dissection, which is felt even more in the anterior resection phase of the rectum with mesorectal excision, and in the dissection of the Denonville fascia from the presacral space, seminal vesicles, and prostate. A particular advantage could be highlighted in the possibility of neurosalvage with preservation of the hypogastric plexus.

Robotic surgery is increasingly being used for more colorectal procedures, but is still rarely used for multiquadrant abdominal resections. A study published in 2019 by Rodriguez et al. comparing 15 robot-assisted (RA) and eight laparoscopic colectomies (LC) reported shorter hospital stays: four days for RA vs. six for LC; less blood loss: 50 ml for RA vs. 100 ml for LC; and less operative time: 243 min for RA vs. 263 min for LC [6].

In 2022, Bianchi et al. published results from 23 retrospective studies, including 736 robotic and 9,904 laparoscopic multiquadrant surgeries [7]. Summary data showed that robotic had a lower conversion rate (OR = 0.17; 95% CI, 0.04-0.82; p = 0.03) than laparoscopic. A lower mean blood loss of ≈65 mL was reported in the robotic group. The robotic approach was associated with longer operative times. No differences for postoperative complications and hospital stay. No reports of urologic outcomes, sexual dysfunction, and quality of life.

Among the disadvantages, we would highlight the longer operating time, 120 to 180 minutes longer operating time compared to the conventional approach. Another disadvantage is the specific preparation of the equipment, which requires specific and in-depth knowledge to ensure the smooth running of the surgical intervention.

## Conclusions

This clinical case provides an important example of the multidisciplinary approach in the diagnosis and treatment of FAP. Neoadjuvant therapy, including radiotherapy and chemotherapy followed by robot-assisted surgery, demonstrates the significant potential of modern treatment approaches. This highlights the importance of personalized treatment in the management of this complex disease. Although neoadjuvant therapy did not result in a complete reduction in one of the tumors, its administration prior to surgical intervention improved operative conditions and contributed to a better outcome. The use of the da Vinci Xi robot-assisted platform allowed for precision and minimal invasiveness, which is essential in complex surgical procedures. The successful management of subcutaneous emphysema in the early postoperative period and the absence of significant complications highlight the effectiveness of postoperative care and the importance of early mobilization and nutrition. These aspects are critical for optimizing recovery and reducing the risk of complications.

Collaboration between gastroenterologists, oncologists, radiologists, and surgeons is essential for the comprehensive management of such complex cases. Finally, this clinical case highlights the need for long-term surveillance of patients with FAP for early detection and management of potential recurrences or new disease. The choice of treatment depends on multiple factors, including the stage of the disease, the presence of comorbidities, and the patient's general health. Robot-assisted proctocolectomy is a relatively new approach whose advantages and disadvantages are yet to be explored.
